# Health service utilization, economic burden and quality of life of patients with mucopolysaccharidosis in China

**DOI:** 10.1186/s13023-024-03333-4

**Published:** 2024-09-06

**Authors:** Qi Kang, Yuhang Fang, Yan Yang, Dingguo Li, Lin Zheng, Xinyi Chen, Xiaowen Tu, Chunlin Jin

**Affiliations:** 1 Shanghai Health Development Research Center (Shanghai Medical Information Research Center), Shanghai, China; 2 Shanghai-MOST Key Laboratory of Health and Disease Genomics, NHC Key Laboratory of Reproduction Regulation, Shanghai Institute for Biomedical and Pharmaceutical Technologies, Shanghai, China; 3 Shanghai Foundation for Rare Disease, Shanghai, China; 4 Beijing Zhengyu MPS Care Center for Assistance, Beijing, China; 5 Putuo Hospital, Shanghai University of Traditional Chinese Medicine, Shanghai, China

**Keywords:** Mucopolysaccharidosis, Diagnoses, Treatment, Economic burden, Health-related quality of life, Rare disease

## Abstract

**Background:**

Patients with mucopolysaccharidosis (MPS) often face delayed diagnoses, limited treatment options and high healthcare costs, that may significantly affect patients' quality of life. The objective of this study was to understand medical service utilization related to diagnosis and treatment, economic burden during diagnosis period, and health-related quality of life among MPS patients in China.

**Methods:**

A series of patients diagnosed with MPS registered in the national patient organization were recruited for a cross-sectional survey from May to July 2019. Information were collected from patients or their parents via phone interview, including demographic data, utilization of services related to diagnosis and treatment, total cost during the period of MPS diagnosis and health-related quality of life (HRQoL). HRQoL was assessed by PedsQL 4.0 Generic Core Scale (PedsQL) and 36-item short-form health survey (SF-36) depending on the age of patients with MPS and compared with the general Chinese population.

**Results:**

A total of 180 MPS patients (50, 67, 15, 46, 1 and 1 for type I, II, III, IV, VI and VII), with a mean age of 9.54 years and 137 (76.11%) males, were included in analysis. The mean age at first visit to a medical doctor for MPS related symptoms was 3.65 ± 2.58 years old, while only 12 patients (6.67%) were diagnosed on their first visit. The mean diagnostic delay, which is defined as the time between the first visit to a medical doctor for MPS related symptoms and the final diagnosis, was 9.42 months, with no significant difference between types. The average number of misdiagnosis was 4.56. Before the confirmed diagnosis, the patients made an average of 6.31 visits and visited 4.3 hospitals. During diagnosis period, the mean of ¥81,086.72 direct medical costs accounted for 63.75% of the total cost. Only 32.78% of the patients had ever received specific treatments. The mean scores of PedsQL and SF-36 of patients were significantly lower than the Chinese norms. Household annual income per person, specific treatment use and MPS subtype were significantly associated HRQoL of patients.

**Conclusion:**

The results highlight challenges faced by MPS patients in terms of diagnosis, access to specific treatments, economic burden and low HRQoL. There is an urgent need to improve early detection and diagnosis, create fair and consistent mechanisms to increase access to specialized treatment and reduce the economic burden of MPS patients in China.

## Introduction

Mucopolysaccharidosis (MPS) is the most common type of lysosomal storage disorders (LSDs) caused by autosomal recessive defects, which are characterized by a natural deficiency in the enzymes that break down glycosaminoglycans (GAGs) [1–3]. Based on the specific enzyme deficiency present, MPS are categorized into seven distinct clinical types, described as MPS I through MPS IX (excluding MPS V and VIII), and some types are further categorized into subtypes [4]. MPS can lead to a range of symptoms and health problems, including skeletal abnormalities, developmental delays, vision and hearing loss, heart problems, and neurological complications [2]. The estimated incidence of MPS ranges from 1.04 to 4.8 in 100,000 live births worldwide, with some types being more common in some populations and ethnic groups due to consanguineous marriages and other factors [4–6]. For instance, the incidence rate of MPS in the United States was 0.98 per 100,000 live births, with the common types being MPS I, II, and III (accounted for 26%, respectively) [7]; while it was 2.04 per 100,000 live births in Taiwan, with MPS II accounted for 52.3%, MPS I accounted for 5.4%, MPS III accounted for 19.2% [8]. Eastern China is also dominated by MPS II (47.4%), followed by MPS IVa (26.8%), MPS I (16.3%), MPS VI (4.2%), MPS III (4.2%) and MPS VII (1.1%) [3].

MPS is a genetic chronic disease that can occur from the fetal period to adolescence. Diagnosis delay in MPS patients is widespread due to lacking early specificity [9–13]. A Dutch study showed that the diagnostic delay for patients with MPS I and MPS III had not shorten between 1988 and 2017 [10]. Patients with delayed diagnosis do not receive timely treatment, which in turn leads to irreversible clinical features, multiple organ damage [4, 14], reduced quality of life [15–17], and increased additional medical costs [17].

The specific therapies of MPS include enzyme replacement therapy (ERT), hematopoietic stem cell transplantation (HSCT), and gene therapy. ERT directly introduces functional enzymes into the body to reduce or normalize GAG levels [18–23]. HSCT and gene therapy are considered permanent treatments. HSCT involves the transplantation of cells from bone marrow, peripheral blood, or umbilical cord blood, which penetrate throughout the body's tissues and organs to produce degrading enzymes to alleviate symptoms [24]; gene therapy is not limited by donor or blood–brain barrier restrictions but may cause rejection of gene products [25]. However, specific treatments are expensive [26, 27] and may be not covered by national health insurance [28, 29], which places a heavy economic burden on patients [30, 31]. As a consequence, the accessibility of specific treatments and health-related quality of life (HRQoL) for patients with MPS would be negatively affected.

Overall, MPS has an early onset in both developed and developing countries, with a long diagnostic delay in most patients [10–12, 32, 33]. After diagnosis, the use of specific treatments such as ERT is low, especially in developing countries [9, 28, 34, 35]. Ultimately, HRQoL of MPS patients is severely impaired, both in physical and psychological, and is much lower than that of the general population [32, 36–38]. However, previous studies have focused on a single type of MPS [9, 32, 34–36, 38–40] or discussed MPS within the broad category of LSDs [28] with small sample sizes. In addition, although studies have suggested that delay in diagnosis increases pre-diagnosis health care costs [17], the exact economic burden is unclear. The objective of this study was to understand medical service utilization, assess the economic burden during diagnosis period and HRQoL, and explore factors influencing HRQoL among MPS patient in China.

## Methods

### Study design

This was a nationwide cross-sectional study aimed to investigate the survival status and disease burden of MPS patients in China. It was based on a convenience sample because MPS patients are scattered and no sampling frame is available in China. All MPS patients that were recruited for the study are registered in Beijing Zhengyu MPS Care Center (BZ-MPSCC) which was established in 2012—the only national patient organization providing services related to medical treatment and rehabilitation to MPS patients. The survey was carried out via phone calls by trained interviewers from Livingston Market Consultants (LMC), an independent market research and consultant company using a structured questionnaire between May and July 2019. The questionnaire was designed by Shanghai Health Development Research Center (SHDRC), BZ-MPSCC and LMC, based on a literature review and interviews with doctors, health economy and policy researchers in the field of rare diseases. Informed consent was obtained from patients and their guardians. The study was approved by the Medical Ethics Committee of SHDRC.

### Inclusion criteria

Patients who met all the following conditions were recruited: (*i*) They were diagnosed in the hospital with specific type of MPS, including type I, II, III, IV, VI or VII; (*ii*) The patients or their primary caregivers were willing to participate in the survey and were able to complete the questionnaire. Among 378 patients registered in BZ-MPSCC, 180 of them completed the survey. Since most of the patients were children and adolescents, 170 questionnaires were answered by their parents. The other 10 patients who were above 21 years old conducted interview with the help from their parents.

### Measurements

#### Health service utilization

The measures of health service utilization covered utilization of service related to diagnosis and treatment including age (year/month) at initial visit and confirmed diagnosis, types of hospital visited as well as number of visits, misdiagnoses, and hospitals visited, treatment after diagnosis (Without any treatments/Only symptomatic treatment, including orthopaedic surgery, heart valve replacement, corneal transplantation, physical therapy, etc./Only specific treatment, including ERT, HSCT and gene therapy/Both symptomatic and specific treatment).

#### Cost during the diagnostic period

In this study, we conducted a retrospective assessment of the total cost during the period of MPS diagnosis, which encompassed direct healthcare costs, direct non-healthcare costs, and indirect costs incurred by patients and their families. Costs comprised the following components: Direct healthcare costs: (i) laboratory and test costs; (ii) treatment and surgical costs; (iii) drugs’ costs; (iv) costs of nursing, rehabilitation or physiotherapy; Direct non-healthcare costs: (v) transportation costs, including expenses for aircraft, trains, taxi, and public transportation; (vi) accommodation costs, including hotels, rents due to visits; (vii) health care, nutritional supplementation; Indirect costs: (viii) daily lost wages; (ix) other indirect costs. For the purpose of comparison, the exchange rate is based on 1 USD ($) = 7 CNY (¥).

#### HRQoL

The PedsQL 4.0 Generic Core Scale (PedsQL), originally developed by Varni, was used to assess HRQoL of children aged 2 to 18 [41]. The Chinese version also has good reliability and validity [42]. The PedsQL scale evaluates HRQoL across four dimensions: physical functioning (8 items), emotional functioning (5 items), social functioning (5 items), and school functioning (5 items). The psychosocial health dimension comprises the emotional, social, and school functioning subscales. For each item, a five-point response scale was used (0 ~ 4, never a problem ~ always a problem). Items were reverse-scored and converted to a hundred mark system (0 = 100, 1 = 75, 2 = 50, 3 = 25, 4 = 0), so that higher scores indicated better HRQOL.

The 36-item short-form health survey (SF-36) was used to evaluate the HRQoL of adolescents aged 16 years and above. The scale was revised by Ware and Sherbourne based on the RAND Corporation's Health Insurance and has been translated, localized, and validated in China [43–45]. The scale includes eight dimensions: physical functioning (PF), role limitations due to physical problems (RP), bodily pain (BP), general health (GH), vitality (VT), social functioning (SF), role limitations due to emotional problems (RE), and mental health (MH). Each dimension scores were calculated by summing across items in the same dimension and convert to a 0–100 scale, with higher scores indicating better health status [46].

### Statistics analysis

Descriptive analysis was carried out using mean and standard deviation (SD) for continuous variables and proportions for dichotomous or categorical variables. Analysis of variance and chi-square tests were used for comparison between groups, and linear regression model was used to explore factors associated with HRQoL. All analysis was carried out with Stata SE 15.0 (StataCorp LLC, College Station, TX, USA).

## Results

### Sample characteristics

The average age of participants with MPS in this study was 9.54 ± 6.02 years, with a majority being younger than 10 years old (63.89%), male (76.11%), and from the eastern (44.59%) and central (33.76%) regions of China. More than seventy percent (72.23%) of them had a household annual income per person of less than ¥20,000 ($2857.14). The most common type of the patients was type II (37.22%), followed by type I (27.78%) and type IVa (25.56%) (Table 1).

**Table 1:** Demographic characteristic of the study participants

Variables	N	%/Mean ± SD
Region		
East	82	45.56
Central	59	32.78
West	34	18.89
Northeast	5	2.78
Sex		
Male	137	76.11
Female	43	23.89
Age (years)	180	9.54 ± 6.02
0 ~	38	21.11
5 ~	77	42.78
10 ~	40	22.22
≥ 15	25	13.89
Annual household income per capita (¥)		
< 10,000	64	35.56
10,000 ~	66	36.67
20,000 ~	19	10.56
≥ 30,000	31	17.22
Type of MPS		
Type I	50	27.78
Type II	67	37.22
Type III/VI/VII*	17	9.45
Type IVa	46	25.56
Total	180	100.00

*Number of type III, type VI and type VII is 15, 1 and 1, respectively

### Diagnosis and treatment service utilizations

As shown in Table 2, the average age at first visit to a medical doctor for MPS related symptoms for type II (4.23 years) and type IVa (4.24 years) patients were more than one year later than type I (3.13 years) and type III /VI/VI patients (3.13 years). The average age at confirmed diagnosis was 4.15 years old (3.59 years, 4.93 years, 5.10 years and 3.27 year for type I, type II, type IVa and type III/VI/VI, respectively). It took 9.42 months on average for the diagnosis to be confirmed, and over 20% of patients took more than one year to be diagnosed, with no significant difference between types. Prior to the confirmed diagnosis, MPS patients made an average of 6.31 visits and visited 4.3 hospitals. The average number of misdiagnosis was 4.56 (5.34, 3.27, 4.60 and 6.60 for type I, type II, type IVa and type III/VI/VI, respectively), with significant difference between types (F = 3.73, *p* = 0.013).

**Table 2:** Diagnosis and treatment service utilizations

Variables	N	%/Mean ± SD	*F/*Χ^*2*^	*p*-value*
Age at first visit (years)	178	3.65 ± 2.58	2.75	0.045
Age at diagnosis (years)	132	4.15 ± 3.22	2.61	0.054
Diagnostic delay (months)	132	9.42 ± 20.98	0.17	0.919
0	12	9.09		
< 1	63	47.73		
1 ~ 6	23	17.42		
7 ~ 12	7	5.30		
> 12	27	20.45		
Diagnosed at first visit	180	6.67	5.07	0.167
Level III pediatric hospital	95	6.32		
Level III general hospital	62	8.06		
Overseas hospitals	3	33.33		
Others	20	0.00		
Number of visits before diagnosed	143	6.31 ± 6.85	0.90	0.445
Number of hospitals before diagnosed	143	4.30 ± 5.59	0.50	0.683
Number of misdiagnoses	143	4.56 ± 4.01	3.73	0.013
Treatments			6.55	0.684
Without any treatments	9	5.00		
Only symptomatic treatment	112	62.22		
Only specific treatment	43	23.89		
Symptomatic & specific treatment	16	8.89		

*Comparison between subtypes

Only one third of the patients (32.78%) had ever received specific treatments: 22.78% of them received HSCT, 11.67% ERT, and 0.56% gene therapy. The main reasons for not receiving ERT were “the costly burden of treatment” (51.59%), “it was not available” (26.75%), “missed optimal treatment timing” (6.37%); and for HSCT were “the costly burden of treatment” (18.98%), “the doctor didn't think it was necessary” (18.98%), “missed optimal treatment timing (17.52%), “worried about the treatment risks” (15.33%), and “worried about the feasibility of the treatment” (11.68%).

### Economic burden during diagnosis period

The average total cost for patients during diagnosis period was ¥127,196.72 ($18,170.96). Overall, direct medical costs accounted for a higher proportion (63.75%) than direct non-medical costs (20.72%) and indirect costs (15.53%). Of the direct cost, the highest cost was nursing/rehabilitation/physical therapy (¥30,335.56/$4333.65, 37.41% of the direct medical costs), and health care/nutrition supplement was the highest direct non-medical costs (¥10,808.33/ $1544.05, 41.02% of the direct non-medical costs). The costs of daily lost wages (¥16,269.44/$2324.21) accounted for 82.34% of the indirect costs (Table 3).

**Table 3:** Costs per person before diagnosis (n = 180)

Variables	Mean ± SD (¥)	% Category of cost	% Total cost
Direct medical costs	81,086.72 ± 122,966.34	100.00	63.75
Medical tests	17,207.22 ± 12,897.76	21.22	13.53
Surgery	15,023.33 ± 40,172.97	18.53	11.81
Drugs	18,520.61 ± 48,665.80	22.84	14.56
Rehabilitation/nursing	30,335.56 ± 56,193.81	37.41	23.85
Direct non-medical costs	26,350.56 ± 31,043.45	100.00	20.72
Transportation	8162.22 ± 11,161.88	30.97	6.42
Accommodation	7380.00 ± 11,208.53	28.01	5.80
Health care/nutrition	10,808.33 ± 18,083.64	41.02	8.50
Indirect costs	19,759.44 ± 30,756.90	100.00	15.53
Daily lost wages	16,269.44 ± 27,091.78	82.34	12.79
Other costs	3490.00 ± 11,277.78	17.66	2.74
Total costs	127,196.72 ± 158,126.60	100.00	100.00

### HRQoL of patients

Table 4 shows that the HRQoL of MPS patients was lower than the Chinese norms [42, 47]. For those aged 2–15, patients had significantly lower score than the Chinese norm in all dimensions (*p* < 0.001) [42]. For those aged ≥ 16, the scores in all dimensions except for GH were also considerably lower than the Chinese national norm (*p* < 0.001) [47].

**Table 4:** Quality of life of MPS patients: comparison to healthy normative sample

Domains	Patients	Normative	*p*-value
PedsQL (2-15Y, n = 158)			
Physical health	29.17 ± 25.02	88.29 ± 11.39	< 0.001
Psychological health	35.17 ± 21.51	82.99 ± 12.70	< 0.001
Emotional functioning	47.03 ± 22.52	79.39 ± 17.71	< 0.001
Social functioning	32.56 ± 25.27	86.94 ± 15.30	< 0.001
School functioning	25.91 ± 24.78	82.71 ± 14.55	< 0.001
Total scale	33.08 ± 20.25	84.94 ± 10.88	< 0.001
SF-36 (≥ 16Y, n = 19)			
Physical function (PF)	35.00 ± 25.66	87.92 ± 16.98	< 0.001
Role physical (RP)	35.53 ± 39.37	77.50 ± 34.86	< 0.001
Bodily pain (BP)	51.32 ± 32.78	82.22 ± 16.98	< 0.001
General Health (GH)	60.00 ± 14.04	62.51 ± 17.88	0.508
Vitality (VT)	52.37 ± 19.25	68.17 ± 17.63	< 0.001
Social function (SF)	41.45 ± 22.84	80.67 ± 19.98	< 0.001
Role emotional (RE)	36.84 ± 42.88	67.86 ± 39.44	< 0.001
Mental health (MH)	53.26 ± 19.23	68.47 ± 16.90	< 0.001

### Factors associated with PedsQL score

Household annual income per person, specific treatment use, and MPS type are important predictors of PedsQL scores. Compared with patients whose household annual income ≥ ¥30,000 per person, PedsQL scores decreased by 14.92 (*p* = 0.003) in patients with annual household income < ¥10,000 CNY/person and by 10.46 (*p* = 0.129) in patients with ¥10,000 ~ 20,000/person. MPS patients who received ERT only (β = 13.15, *p* = 0.028) or HSCT/gene therapy (β = 10.16, *p* = 0.009) had significantly higher PedsQL scores than those who did not receive specific treatments. In term of types of MPS, the PedsQL scores of Type I (β = − 13.07, *p* = 0.003) and Type III/VI/VII (β = − 11.57, *p* = 0.045) were significantly lower than those of Type IVa. Age, gender, and region were not significantly associated with PedsQL scores (Table 5).

**Table 5:** Factors associated with PedsQL score (n = 156)

Variables	*β* (95% CI)	SE	*Z*	*p*-value
Region				
Central	− 2.36 (− 9.39, 4.67)	3.59	− 0.66	0.511
West	0.81 (− 7.72, 9.34)	4.35	0.19	0.852
Northeast	− 14.34 (− 33.67, 4.99)	9.86	− 1.45	0.146
East	Ref			
Sex				
Male	− 2.22 (− 10.06, 5.63)	4.00	− 0.55	0.580
Female	Ref			
Age (Year)				
2 ~	− 1.92 (− 11.10, 7.27)	4.68	− 0.41	0.683
5 ~	0.72 (− 6.38, 7.82)	3.62	0.20	0.843
10 ~	Ref			
Average household income (￥)				
< 10,000	− 14.92 (− 24.71, − 5.13)	5.00	− 2.99	0.003
10,000 ~	− 10.46 (− 19.87, − 1.05)	4.80	− 2.18	0.029
20,000 ~	− 9.85 (− 21.80, 2.09)	6.09	− 1.62	0.106
≥ 30,000	Ref			
MPS subtype				
Type I	− 1.52 (− 10.22, 7.19)	4.44	− 0.34	0.733
Type II	− 13.21 (− 21.54, − 4.89)	4.25	− 3.11	0.002
Type III/VI/VII	− 15.29 (− 26.05, − 4.54)	5.49	− 2.79	0.005
Type IVa	Ref			
Treatment				
Only ERT	13.15 (1.42, 24.89)	5.99	2.20	0.028
HSCT/Gene therapy	10.16 (2.59, 17.74)	3.87	2.63	0.009
Without specific treatment	Ref			

## Discussions

This is a large sample study to profile the medical service utilization, disease burden and HRQoL of patients with all subtypes of MPS in China. The findings of this study show challenges faced by patients with MPS in China, including diagnostic delay, limited access to specific treatments, high economic burden prior to confirmed diagnosis and low HRQoL.

MPS has an early age of onset [9–11, 14, 32–35, 38, 39]. In this study, the mean age at first visit to a medical doctor for MPS related symptoms was 3.65 years. However, obvious diagnosis delay is observed in this study. The mean age at confirmed diagnosis was 4.15 years, which was 9.4 months on average from the first visit to a medical doctor. The length of diagnosis delay in this study (median: < 1 months) is shorter than that reported in Indian MPS patients (median: 39.5 months) [11], and Dutch MPS type I and type III patients (type I median: 9 months, type III median: 33 months) [10]. One reason might be that parents of the patients in the study were more active in seeking health services. The other reason might be that the waiting time to see a specialist in Chinese health service system is shorter because patients in China use self-scheduling services in a hospital to make an appointment but not referral services between all levels of the health system used in other countries [48].

The possible reason for the diagnostic delay is that doctors lack awareness of rare diseases [11, 49]. Among patients diagnosed at first visit in this study, all of them were diagnosed in level III hospitals located at provincial capitals and municipalities (e.g., Beijing and Shanghai). However, even in level III hospitals, the proportion of diagnosed at first visit was only 7%. In this study, the average number of misdiagnosis for MPS patients was more than 4 times. Patients had to visit more than 4 hospitals to have the final diagnosis, which further increases the time period to confirmed diagnosis and leads to a high pre-diagnosis economic burden. Kuiper et al. reported that the diagnostic delay was shorten after first visits to medical specialist but not general practitioner [10].

We fail to find any study that reported the cost for MPS patients during diagnosis period. In this study, the average per person cost for diagnosis was as high as ¥127,196.72 ($18,170.96), which is higher than the per capita disposable income of Chinese residents in 2022 (¥36,883/$5,179.47) [50]. It is worth noting that to improve the diagnosis and treatment capabilities and ensure the accessibility of drugs for rare diseases in China, a three-level National Network to Collaborate on Diagnosis and Treatment of Rare Diseases (NCDTRD) was established by the Chinese government in 2019, comprising 324 hospitals nationwide, including 1 leading national institution, and 32 leading provincial institutions [49], which will be helpful to improve diagnostic delays for Chinese MPS patients. Based on the experiences from other countries, further actions should be taken by government and medical community, including building regional specialistic centers of LSD or MPS, strengthening multidisciplinary diagnosis and treatment, improving diagnosis and treatment guidelines and educating specialists [51].

Specific treatments utilization for MPS patients in this study (32.78%) was lower than that reported in European MPS VII patients (53.8%) [9] and American MPS I patients (44%) [35]. The main reasons for low utilization of specialized treatment were low availability and affordability. Although many ERT drugs for MPS was on the market in US, EU and Japan before 2019, only two drugs were included in *the List of the First Batch of Overseas New Drugs Urgently Needed in Clinical Settings* issued by the Center for Drug Evaluation (CDE) of the National Medical Products Administration (NMPA) in November 2018 and only one drug was authorized for marketing in May 2019 in China [46]. According to the 2022 China Rare Disease Industry Observation Report, the annual ERT costs range from ¥450,000 ($64,285.71) to ¥2,300,000 ($328,571.43) per person [27]. None of the ERT drugs for MPS has been included in the National Health Insurance Reimbursement Catalog (NHIRC), however, and only a few provinces or municipalities have formulated reimbursement policy for expensive ERT drugs for rare diseases [27, 28]. For example, Shanghai Municipality had included ERT (MPS I/II/VI) and HSCT in the Shanghai Children's Hospitalization Mutual Aid Fund since 2011, reimbursing ¥100,000/year per person [52]; and launched a citywide commercial supplemental medical insurance (one-year period) since 2021, reimbursing ERT drugs for MPS II/IVa at 70%, with a reimbursement ceiling of ¥1,000,000/person [53]. In addition, unaffordable out-of-pocket costs after reimbursement, difficulties and delays in reimbursement processes, and limited financial assistance from government or charity continue to prevent MPS patients from accessing specific treatments [28, 31, 51]. With the exception of some developed countries, more and more developing countries have included ERT in the national reimbursement system [54–56]. Further efforts are urgently needed to increase availability and affordability to specialized treatment for MPS patients by government and society in China.

MPS is a complex, progressive, and multi-system LSD. Due to diagnostic delay and unaffordability of high medical costs, about 67% of Chinese MPS patients have difficulty in controlling disease progression and develop irreversible symptoms such as skeletal deformities, hepatosplenomegaly, sensory deficits, and cardiac disease, significantly reducing their HRQoL [4, 9]. Not surprisingly, the HRQoL of MPS patients was significantly lower than that of the general population for almost all dimensions of the PedsQL or SF-36 covering physiological, psychological, and social aspects in this study, which is in line with other studies [33, 39]. The low HRQOL in MPS patients might attribute to multiorgan involvement and trajectory of cognitive decline in some types of MPS. The low score on physiological dimensions (i.e., difficulty doing daily activity, physical pain, and low energy) is likely a direct consequence of the disease manifestations, while the low score on psychological and social dimensions may be impacted on a secondary level for MPS patients [34]. In terms of social function, for example, Meijer [57] found that physical limitations and pain in chronically ill children were associated with a lower level of social activities. Besides, differences in physical appearance and basic self-care and communication skills between MPS patients and their healthy peers could lead to teasing or social isolation [34], which would have further negative impact on their social function.

Consistent with the findings from previous rare disease studies [58, 59], we found that low household economic level was significantly associated with lower HRQoL of MPS patients in this study. The reason might be that higher-income families have more access to social support, quality healthcare and specialty care [58, 59]. Efforts to improve the household economic status of MPS patients would guarantee better HRQoL for them. Similar to findings from other rare disease studies [60, 61], significant differences in HRQoL between subtypes of MPS was observed in this study. Compared with MPS IVa, MPS II and III/VI/VII patients were more likely to have low HRQoL. According to previous studies, the number and severity of disease symptoms is negative associated with HRQoL [39]. The difference in disease severity and cognitive impairment between subtypes might be contributed to type differences in HRQoL. For example, MPS III patients tend to have severe neurological system lesions with rapidly progressive symptoms including cognitive decline, idiopathic developmental delay, hyperactivity, and sleep disorder [4, 62], patients with MPS VI present with severe clinical symptoms in the neonatal period [63], and patients with the severe form of MPS II also present central nervous symptoms rapidly and tend to die in the first decade of life without effective treatment [4].

In line with previous studies [32, 34], we found that specific therapeutic use was associated with higher HRQoL. ERT and HSCT can normalize the concentration of GAGs in the target organ and improve organomegaly, pain, airway obstruction, corneal clouding, hearing, and motor function. Clinical trials have demonstrated the efficacy of ERT and HSCT for MPS I, II, IV, VI, and VII, with better outcomes for a combination of HSCT and ERT [4, 64]. Unfortunately, both HSCT and ERT have proven unsatisfactory in patients with MPS III because MPS III is a special type of MPS that primarily affects central nervous system. Gene therapy is important options for MPS III to improve outcomes. However, such specific treatments do not affect irreversible lesions such as short stature, neurological lesions, and vascular malformations [64, 65]. Early diagnosis and early initiation of specific treatment is believed to be key in improving clinical- and patients-reported outcomes and patient quality of life [2, 66], especially for subtypes with rapid progression. During treatment period, it is essential for MPS patients to timely receive adequate ERT to improve symptom and prolong life.

In addition, rehabilitation for MPS patients played an important role in managing disabilities and quality of life [2, 56, 67, 68]. Dusing et al. recommended that children diagnosed with MPS type I be referred to rehabilitation services at the time of diagnosis [69]. Cibele et al. recommended that otorhinolaryngologist should be part of the group of professionals that follows MPS patients to better monitor their hearing and provide early hearing rehabilitation [70]. Because of several serious complications, a multidisciplinary treatment and rehabilitation team, life-long therapies and life-cycle management of patients with MPS should be implemented and emphasized.

### Limitations

Firstly, the study was based on a convenience sample due to lack of a national statistical surveillance network. Thus, the findings may not be generalized to all. However, this is the largest sample study covering all types of MPS in China. Secondly, self-reported data obtained through a questionnaire on health service utilization and cost during the diagnostic period are inevitably subject to recall bias. Thirdly, due to small sample size, we failed to compare MPS type III, VI and VII individually and explore factors associated with HRQoL among patients over 15 years, which needs to be further explored in the future. Finally, results from this cross-sectional study could not provide evidence for causality but only for associations.

### Conclusion

Diagnostic delay, high economic burden and limited access to specific treatments are associated with low HRQoL of MPS patients and are common challenges faces by MPS patients in China. Findings of this study highlight the need to improve early detection and diagnosis, create fair and consistent mechanisms to increase access to specialized treatment, reduce the economic burden and improve HRQoL of MPS patients in China. In addition to strengthen training and cooperation among the NCDTRD**,** which play a key role in addressing rare disease issues in diagnosis, management and treatment,** s**ubstantial efforts are urgently needed to deal with the availability and affordability of specialized treatment for MPS patients. For example, improving perinatal genetic screening for rare diseases and including related costs in maternity insurance, developing multi-party healthcare payment schemes and establishing a special fund with the participation of government, commercial companies, charities and patients, encouraging donated drug projects, and setting-up a differentiated registration and approval system for orphan drugs, increase public and government awareness of rare diseases.

## Data Availability

This is an open access journal, and articles are distributed under the terms of the Creative Commons Attribution-NonCommercial-ShareAlike 4.0 License, which allows others to remix, tweak, and build upon the work non‑commercially, as long as appropriate credit is given and the new creations are licensed under the identical terms.
